# 2178. Resistance to Cefepime/Taniborbactam in NDM-1 -Are We Losing the Battle against MDROs?

**DOI:** 10.1093/ofid/ofad500.1800

**Published:** 2023-11-27

**Authors:** Daisuke Ono, Maria F Mojica, Christopher R Bethel, Yoshikazu Ishii, Diego Moreno, Alejandro J Vila, Robert A Bonomo

**Affiliations:** Case Western Reserve University, Brecksville, Ohio; Case Western Reserve University, Brecksville, Ohio; Louis Stokes Cleveland Department of Veterans Affairs Medical Center, Cleveland,, Cleveland, Ohio; microbiology and infectious disease, ota-ku, Tokyo, Japan; IQUIR, Instituto de Química de Rosario, CONICET, Universidad Nacional de Rosario, Rosario, Santa Fe, Argentina; Instituto de Biología Molecular y Celular de Rosario (IBR), Rosario, Santa Fe, Argentina; Louis Stokes Cleveland Department of Veterans Affairs Medical Center, Cleveland,, Cleveland, Ohio

## Abstract

**Background:**

Taniborbactam **(**TAN; **Fig 1**) is a novel cyclic boronate that inhibits Serine- and some Metallo-β-Lactamases like NDM and VIM. The combination cefepime (FEP)/ TAN has just completed a Phase III clinical trial. Notably, resistance mechanisms of FEP/TAN have already been described, such as the one provided by NDM-9 (Glu152Lys; PMID:36796395). The crystal structure of the NDM-1:TAN complex revealed that Lys224 anchors TAN by forming two hydrogen bonds (PMID:31454231). We previously showed that the Arg228Leu substitution in VIM-24, the homologous residue of Lys224 in VIM-2, improves the activity towards ceftazidime (CAZ) and FEP (PMID:25915520 ). Therefore, we hypothesized that substitutions at position 224 in NDM-1 could also have a similar effect and potentially confer FEP/TAN resistance.

**Figure:**
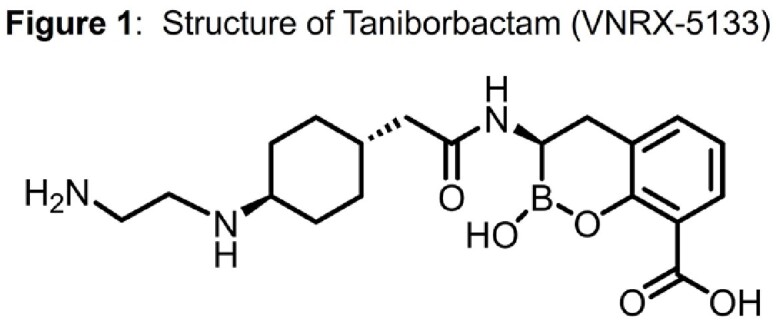

**Figure:**
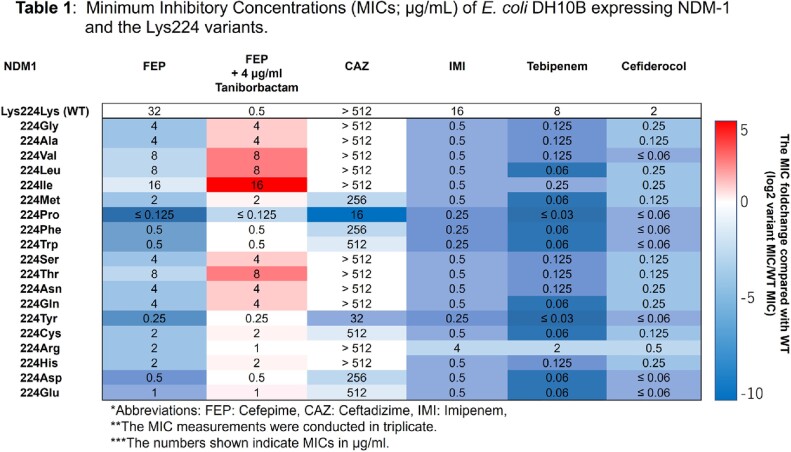

**Methods:**

NDM-1 clones containing all 19 amino acid variants at position 224 were synthesized (Genscript, NJ) and used to transform *E. coli* DH10B. Minimal inhibitory concentrations (MICs) were determined using the CLSI-approved agar dilution method. NDM-1 and Lys224Ile were further studied by steady-state kinetics, inhibitor analysis, and dockings.

**Results:**

Compared to NDM-1, all but the Lys224Ile variant display lower MICs for FEP, CAZ, and imipenem (IMI) and higher MICs for FEP/TAN. Contrary, resistance to FEP and CAZ conferred by the Lys224Ile variant was similar to that by NDM-1. However, TAN did not alter MIC for FEP (**Table 1**). Kinetic assays confirmed differences in the catalytic efficiency of NDM-1 and the Lys224Ile variant to FEP and IMI, respectively (**Table 2**). Likewise, the IC_50_ of TAN for NDM-1 (0.01 µM) was significantly lower than for the Lys224Ile variant (0.14 µM) (**Table 2**). Lastly, docking simulations revealed that the Lys224Ile substitution results in the loss of the anchoring H-bonds and yields an unproductive binding of TAN (**Fig 2-a, b**).

**Figure:**
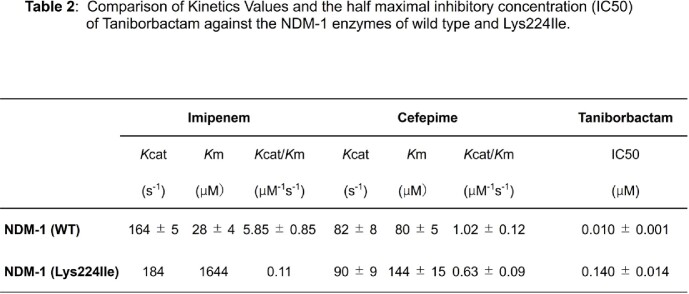

**Figure:**
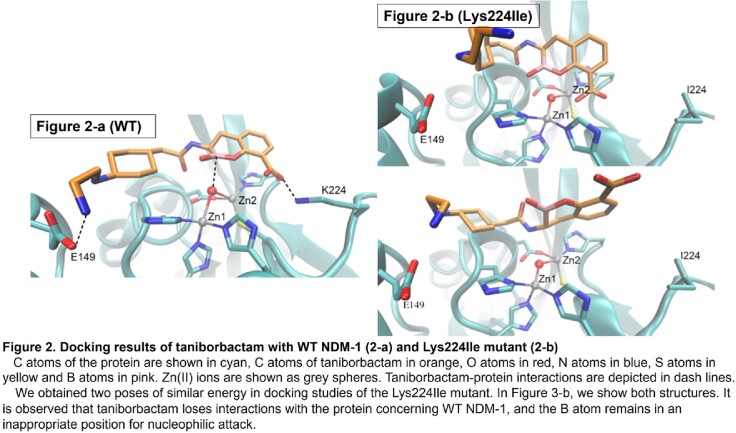

**Conclusion:**

Resistance to CEF/TAN arises from the Lys224Ile substitution in NDM-1. This finding, in addition to the occurrence of NDM-9 (Glu152Lys), may portend vulnerabilities in this novel β-lactam/β-lactamase inhibitor combination by single amino acid changes even before clinical release. Further attention should be focused on exploring the impact of other amino acid substitutions on susceptibility to CEF/TAN to anticipate clinical failure.

**Disclosures:**

**Robert A. bonomo, MD**, Entasis, Merck, VenatoRx, Wockhardt: Grant/Research Support

